# Synthesis and crystal structures of 5,5′-(propane-2,2-di­yl)bis­(2-hy­droxy­benzaldehyde) and 5,5′-(propane-2,2-di­yl)bis­(2-hy­droxy­isophthalaldehyde)

**DOI:** 10.1107/S2056989018016316

**Published:** 2018-11-22

**Authors:** Rosario C. Sausa, Dominika N. Lastovickova, John J. La Scala

**Affiliations:** a US Army Research Laboratory, RDRL-WML-B, Aberdeen Proving Ground, MD 21005, USA; b US Army Research Laboratory, RDRL-WML-G, Aberdeen Proving Ground, MD 21005, USA

**Keywords:** crystal structure, synthesis, photoluminescence, 5,5′-(propane-2,2-di­yl)bis­(2-hy­droxy­benzaldehyde), 5,5′-(propane-2,2-di­yl)bis­(2-hy­droxy­isophthalaldehyde), NMR

## Abstract

The title compounds 5,5′-(propane-2,2-di­yl)bis­(2-hy­droxy­benzaldehyde), C_17_H_16_O_4_, (**1**), and 5,5′-(propane-2,2-di­yl)bis­(2-hy­droxy­isophthalaldehyde), C_19_H_16_O_6_, (**2**), crystallize with one mol­ecule in the asymmetric unit. In mol­ecule (**1**), a >C(CH_3_)_2_ group bridges two nearly planar salicyl­aldehyde groups [r.m.s deviations = 0.010 (1) and 0.025 (2) Å], each comprising a planar phenyl ring bonded with a hydroxyl and an aldehyde group. Similarly, compound (**2**) has the same bridging group, but it connects two nearly planar appendants [r.m.s deviations = 0.034 (1) and 0.035 (1) Å], each comprising a phenyl ring bonded with a hydroxyl and two aldehyde groups. Mol­ecule (**1**) exhibits a bridge angle of 109.5 (2)° with the salicyl­aldehyde planes subtending a dihedral angle of 88.4 (1)°. In contrast, mol­ecule (**2**) presents a bridge angle of 108.9 (2)° with its appendants subtending a dihedral angle of 79.6 (3)°. Both mol­ecules exhibit two intra­molecular O—H⋯O hydrogen bonds involving the phenolic H atoms and carboxyl O-atom acceptors. In the crystal of (**2**), O—H⋯O hydrogen bonds between one of the hydroxyl H atoms and a carboxyl O atom from a symmetry-related mol­ecule form a chain along [10]. In addition, (**2**) exhibits a strong visible luminescence when excited with ultraviolet radiation.

## Abstract 2

The mol­ecule of (**1**) presents a >C(CH_3_)_2_ group that bridges two nearly planar salicyl­aldehyde groups, each comprising a planar phenyl ring bonded with a hydroxyl and an aldehyde group. Similarly, mol­ecule (**2**) presents the same bridging group, but it connects two nearly planar appendants, each comprising a phenyl ring bonded with a hydroxyl and two aldehyde groups. Compound (**2**) exhibits a strong visible luminescence when excited with ultraviolet radiation.

## Chemical context   

As polymers play an undeniable role in our everyday lives, extensive resources and safety evaluations are devoted toward the development and marketing of the most suitable and effective polymer species for a given application (Andrady & Neal 2009 ▸; Fenichell 1996 ▸; Teegarden 2004 ▸). Bisphenols, salicyl­aldehydes, and their derivatives have fueled much inter­est in recent years because they are key precursors for many present and future compounds. Bisphenols typically serve as scaffolds for producing thermoplastics and polymer resins, whereas salicyl­aldehydes and derivatives are commonly used to synthesize metal-chelating agents for analytical, biological, or material science applications (Lim & Tanski, 2007 ▸; Guieu *et al.*, 2012 ▸, 2013 ▸; Barba & Betanzos, 2007 ▸; Vančo *et al.*, 2005 ▸; Baisch *et al.*, 2017 ▸; Kalinowski & Richardson, 2005 ▸, Mounika *et al.*, 2010 ▸). As part of our ongoing work on the synthesis and characterization of novel compounds, as well as our effort to eliminate or replace toxic reagents with greener chemicals in the polymer production process, we have synthesized the title compounds, 5,5′-(propane-2,2-di­yl)bis­(2-hy­droxy­benzaldehyde (**1**) and 5,5′-(propane-2,2-di­yl)bis­(2-hy­droxy­isophthalaldehyde (**2**). These precursor compounds present a >C(CH_3_)_2_ group that bridges two salicyl­aldehyde moieties (**1**) or two phenyl groups with an hydroxyl and two aldehyde appendants (**2**). The various functional groups in these mol­ecules determine their chemical and physical properties, and the ability to modify them provides the title compounds with a wide versatility and the multifunctionality required for synthesizing safer and better performance materials for future civilian and military applications. For instance, the title compounds may be used for the non-toxic, iso­cyanate-free synthesis of polyurethanes (Maisonneuve *et al.*, 2015 ▸). In addition, (**2**) is a new, solid-state photoluminescence material that emits radiation in the spectroscopic range between 490 and 590 nm upon ultraviolet light excitation, with potential use as an organic light emitting diode, laser frequency harmonic generator, or photoelectric converter.
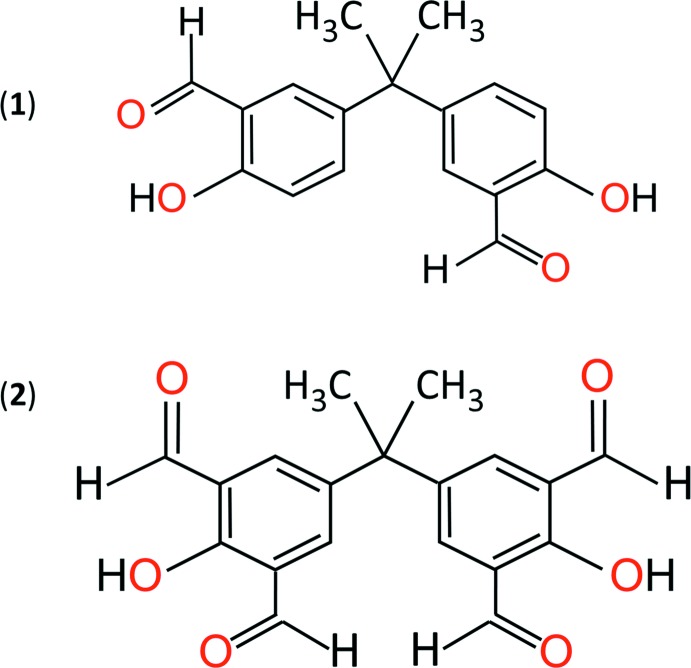


## Structural commentary   

**Figure 1:**
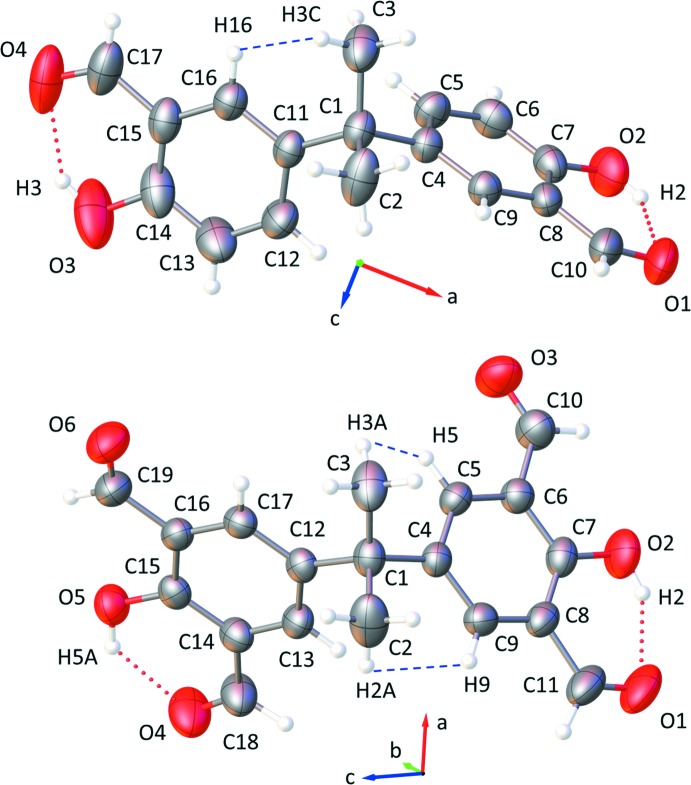
Mol­ecular conformation and atom-numbering scheme for mol­ecules (**1**) (top) and (**2**) (bottom). Non-hydrogen atoms are shown with 50% probability displacement ellipsoids.

Both title compounds have one mol­ecule in their asymmetric unit. Their mol­ecular structures (Fig. 1 ▸) typify bis­phenols and salicyl­aldehyde derivatives, and their bond lengths and angles are in the usual ranges (Lim & Tanski, 2007 ▸; Guieu *et al.*, 2012 ▸, 2013 ▸; Eriksson & Eriksson, 2001 ▸; Barba & Betanzos, 2007 ▸; Vančo *et al.*, 2005 ▸; Baisch *et al.*, 2017 ▸). In the mol­ecule of (**1**), the salicyl­aldehyde fragment containing atom C4 (S1*A*) is near planar [r.m.s. deviation = 0.010 (1) Å], with a maximum out-of-plane deviation of 0.020 (2) Å for the O1 atom. Similarly, its companion salicyl­aldehyde fragment (S1*B*) is near planar [r.m.s. deviation = 0.025 (2)], with a maximum out-of-plane deviation of 0.050 (2) Å for the O3 atom. The bridge angle C4—C1—C11 measures 109.5 (2)° and the S1*A* and S1*B* planes subtend a dihedral angle of 88.4 (1)°. Mol­ecule (**1**) exhibits two intra­molecular hydrogen bonds between the phenolic hydrogen atoms and carboxyl O-atom acceptors (Table 1 ▸).

Mol­ecule (**2**) presents two near planar appendants, denoted A1 and A2 for the appendants containing C4 and C11, respectively, [r.m.s deviation = 0.034 (1) Å (A1) and 0.035 (1) Å (A2), with maximum out-of-plane deviations of 0.068 (2) Å for atom O2 (A1) and −0.060 (2) Å for atom O5 (A2)]. Each appendant comprises a hydroxyl and two aldehyde groups. Similar to (**1**), the salicyl­aldehyde fragments with atoms C4–C9/C11/O1/O2 (S2*A*) or C12–C17/C18/O6/O5 (S2*B*) in (**2**) adopt a near planar geometry [r.m.s. deviation = 0.024 (1) Å for S2*A* and 0.036 (1) Å for S2*B*]. The additional carbonyl groups C10—O3 and C18—O4 on the phenyl rings are twisted slightly out of the S2*A* and S2*B* planes, respectively, as evidenced by their respective torsion angles C5—C6—C10—O3 [−2.9 (4)°] and C13—C14—C18—O4 [−179.1 (3)°]. These additional groups increase the steric hindrance between the appendants and methyl bridge groups in (**2**), perhaps decreasing both the bridge angle C4—C1—C12 [108.9 (2)°] and the dihedral angle between the A1 and A2 planes [88.4 (1)°] relative to (**1**). Mol­ecule (**2**) presents two intra­molecular hydrogen bonds involving the phenolic hydrogen atoms with the carboxyl O-atom acceptors (Table 2 ▸), similar to (**1**).

**Figure 2:**
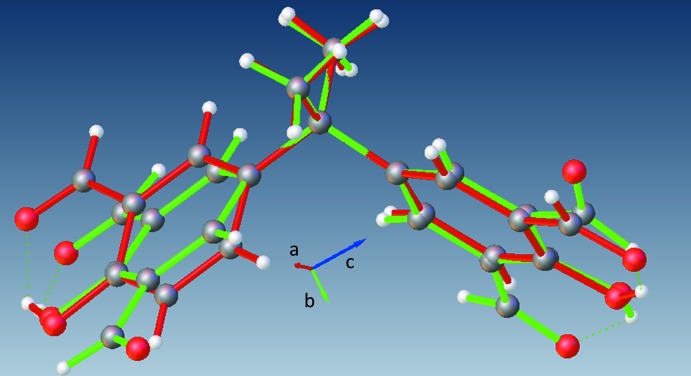
An overlay of (**1**) (red) and (**2**) (green), where the atoms C1/C2/C3/C4/C11 of (**1**) are superimposed with the corresponding atoms of (**2**).

Both (**1**) and (**2**) exhibit several intra­molecular H⋯H contacts that are shorter than the sum of the H-atom van der Waals radii. These contacts occur between the methyl group H atoms and adjacent phenyl group H atoms [H3*C*⋯H16 = 2.2538 (1) Å, shortest in (**1**); H3*A*⋯H5 = 2.1643 (1) Å and H2*A*⋯H9 = 2.1890 (1) Å, shorter than others in (**2**)]. Superimposition of the atoms C1/C2/C3/C4/C11 of (**1**) with the corresponding atoms of (**2**) (see Fig. 2 ▸), yields an r.m.s. deviation of 0.011 Å with the S1*A* and S2*B* planes subtending a dihedral angle of 9.82 (4)° and the S1B and S2*B* planes subtending an angle of 35.1 (1)°.

## Supra­molecular features   

**Figure 3:**
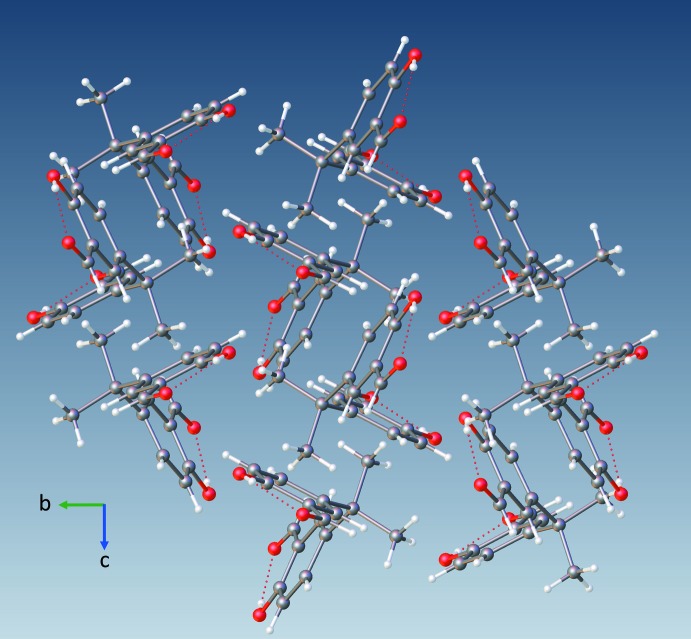
Crystal packing of (**1**) along the *a* axis. Red dashed lines show the intra­molecular O—H⋯O hydrogen bonds.

**Figure 4:**
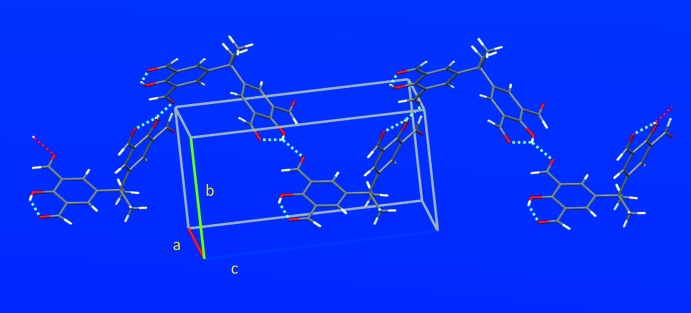
Hydrogen bonding of (**2**) showing both its intra- and inter­molecular hydrogen bonds, depicted as blue dashed lines.

Fig. 3 ▸ shows the packing of (**1**) along the *a* axis. van der Waals contacts between the O atoms and H atoms of adjacent mol­ecules [O1⋯H3*B* ^i^ = 2.628 Å; symmetry code: (i) 1 − *x*, 1 − *y*, −*z*] dominate the inter­molecular inter­actions. In addition, bifurcated contacts between atom C17 and atoms H3 and O3 of adjacent mol­ecules [C17⋯H3^ii^ = 2.887 Å; C17⋯O3^ii^ = 2.811 (4) Å; symmetry code: (ii) *x*, ½ − *y*, −½ + *z*] contribute to the crystal packing. As in mol­ecule (**1**), O⋯H contacts play a key role in the inter­molecular inter­actions of (**2**). However, unlike (**1**), these inter­actions result mostly from hydrogen bonding between the phenolic hydrogen atoms and the carboxyl oxygen atoms of adjacent mol­ecules [O5—H5*A*⋯O1 = 2.841 (2) Å; θ = 131 (3)°; Table 2 ▸.) As a result, each mol­ecule becomes both a hydrogen-bond donor and acceptor. This feature links a mol­ecule at both ends with its adjacent inverted mol­ecules, thus forming undulating chains along [10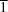] (Figs. 4 ▸ and 5 ▸).

## Database survey   

A search of the Cambridge Structural Database (CSD web inter­face, August 2018; Groom *et al.*, 2016 ▸) and the Crystallography Open Database (Gražulis *et al.*, 2009 ▸) yields a number of compounds containing the bis­phenol or salicyl­aldehyde group. For examples, see Lim & Tanski, 2007 ▸; Guieu *et al.*, 2012 ▸, 2013 ▸; Eriksson & Eriksson, 2001 ▸; Barba & Betanzos, 2007 ▸; Vančo *et al.*, 2005 ▸; Baisch *et al.*, 2017 ▸. The compounds 4-[2-(4-hy­droxy­phen­yl)propan-2-yl]phenol (**3**), a common chemical known also as bis­phenol A, (Lim & Tanski; CCDC 617706, CEGYOC03) and 5-[(3-formyl-4-hy­droxy­phen­yl)meth­yl]-2-hy­droxy­benzaldehyde (**4**) (Barba & Betanzos, 2007 ▸; CCDC 642298, VILCID) merit comparison to (**1**) and (**2**) and further discussion. Mol­ecule (**3**) presents a submolecular structure of the title compounds, as it only lacks the aldehyde groups found in (**1**) or (**2**). In contrast, (**4**) exhibits a pair of salicyl­aldehyde groups as (**1**) or (**2**), except that they are linked by a >CH_2_ bridge, instead of a >C(CH_3_)_2_ bridge.

**Figure 6:**
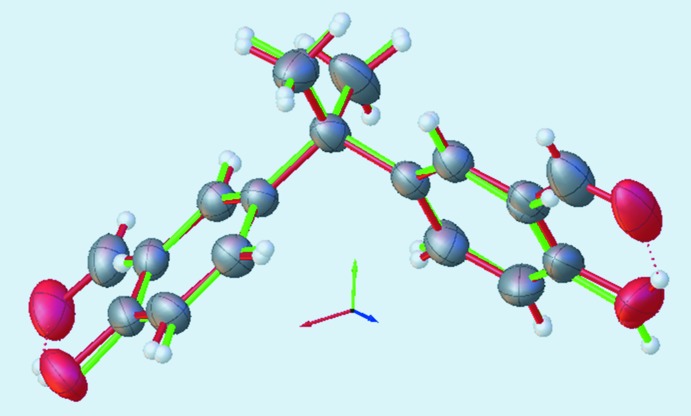
Superimposition of the non-hydrogen atoms of (**3**) (green) onto the corresponding atoms of (**1**) (red).

Compound (**3**) crystallizes with three independent mol­ecules in the asymmetric unit. Each mol­ecule presents a pair of planar phenol fragments [r.m.s. deviations = 0.013 (2) and 0.028 (2) Å; 0.0039 (4) and 0.0078 (5) Å; and 0.0055 (6) and 0.0039 (3) Å] subtending dihedral angles of 77.81 (3), 86.15 (4) and 84.34 (4)°, respectively, and respective bridge angles of 109.2 (1), 109.5 (1), and 108.1 (1)°. In general, both (**1**) and (**2**) have similar geometric parameters to (**3**), although their corresponding phenol groups are less planar than those of (**3**). This manifestation results most likely because the phenyl groups of the title compounds contain aldehyde groups in addition to the hydroxyl groups. The O atoms of these aldehyde groups participate in hydrogen bonding with the hydroxyl H atoms, thus partially displacing the hydroxyl O atoms away from the phenol planes. A superimposition of the atoms in (**1**) with the corresponding atoms of one of the three structures of (**3**) shows that the differences in the atom positions of the two structures are hardly discernible (Fig. 6 ▸) [r.m.s. deviation = 0.115 Å; maximum displacement = 0.217 (2) Å between the O2 atom of (**1**) and its counterpart of (**3**)]. An overlay of structure (**1**) onto either structure two or three of (**3**) yields comparable results. A similar analysis of structures (**2**) and (**3**) yields a r.m.s. deviation of 1.14 Å with maximum displacement of 0.605 (2) Å for the C6 atom of (**2**) and its counterpart in (**3**). Again, we obtain comparable results overlaying either structure two or three of (**3**) onto (**1**).

Mol­ecule (**4**) exhibits a pair of near planar salicyl­aldehyde fragments [r.m.s. deviation = 0.0153 (2) and 0.0238 (9) Å] forming a dihedral angle of 85.96 (4)°, similar to (**1**). Its bridge angle of 113.6 (1)° is much greater than that of (**1**) or (**2**), however. A superimposition of the salicyl­aldehyde group atoms of (**4**) (C4 through C9, C10, O1, and O2) with corresponding atoms of (**1**) reveals nearly identical atomic positions of the two groups [r.m.s. deviation = 0.0160 Å], with the companion salicyl­aldehyde group planes [centroid-to-centroid distance measuring = 4.68 (2) Å] subtending a dihedral angle of 6.81°. A similar analysis for structures (**2**) and (**4**) yields a r.m.s. deviation = 0.027 Å with companion salicyl­aldehyde groups planes [centroid-to-centroid distance measuring = 4.21 (1) Å] forming a dihedral angle of 7.4 (1)°.

## Synthesis and crystallization   

The title compounds were synthesized following modified literature procedures (Özdemir *et al.*, 2015 ▸ and Masurier *et al.*, 2008 ▸ for compounds (**1**) and (**2**), respectively).

Compound (**1**): A combination of compound (**3**) (10.0 g, 43.8 mmol, 1.0 equiv.), paraformaldehyde (16.7 g, 556.1 mmol, 12.7 equiv.), and magnesium(II) chloride (35.2 g, 173.1 mmol, 4.0 equiv.) were suspended in tetra­hydro­furan (THF, 500 mL), placed under a stream of N_2_, and stirred. Then, tri­ethyl­amine (49 mL, 351.6 mmol, 8.0 equiv.) was added dropwise to the reaction mixture at ambient temperature and stirred under reflux for 16 h. At the conclusion of the reaction, the mixture was cooled to room temperature before the addition of diethyl ether (500 mL). The organic solution was sequentially extracted with aqueous 1 *M* HCl (3 × 500 mL) and water (3 × 500 mL), dried over Na_2_SO_­4_ or MgSO4, filtered, and the volatiles were removed under reduced pressure. The solid residue was purified with a series of hexane washes and then dried under vacuum to afford the desired product (**1**) as a white solid (11.3 g, 39.7 mmol, 91% yield). Slow diffusion of hexa­nes into a benzene solution saturated with (**1**) afforded single crystals of (**1**).

Compound (**2**): A mixture of (**3**) (10.0 g, 43.8 mmol, 1.0 equiv.) and hexa­methyl­ene­tetra­mine (19.1 g, 183.3 mmol, 4.2 equiv.) was dissolved in tri­fluoro­acetic acid (TFA, 60 mL) under ambient conditions. The reaction mixture was stirred at 403 K for 2.5 h and subsequently cooled to room temperature before aqueous HCl (3M, 150 mL) was added slowly. The reaction mixture was stirred at 383 K for 16 h, cooled to room temperature, and the resulting organic phase extracted with di­chloro­methane (DCM, 3 × 150 mL). Then, this organic phase was dried over MgSO_4_, filtered, and the volatiles were removed under reduced pressure. The resulting solid was purified with a series of hexa­nes washes and dried under vacuum to afford the novel product (**2**) as a neon yellow solid (9.97 g, 29.3 mmol, 67% yield). Slow evaporation of a DCM solution saturated with (**2**) afforded single crystals suitable for X-ray diffractometry.

Nuclear magnetic resonance (NMR) spectra were recorded on a Bruker 400 MHz spectrometer. Chemical shifts (δ) are given in ppm: (**1**) ^1^H NMR (CDCl_3_, 400.13 MHz): δ 1.70 (*s*, 6H), 6.92 (*d*, *J* = 8.7 Hz, 2H), 7.35 (*dd*, *J*_1_ = 8.7 Hz, *J*_2_ = 2.5 Hz, 2H), 7.43 (*d*, *J* = 2.5 Hz, 2H), 9.86 (*s*, 2H), 10.93 (*s*, 2H) ppm. ^13^C NMR (CDCl_3_, 100.62 MHz): δ 30.47, 41.86, 117.85, 120.15, 130.92, 136.20, 141.67, 160.10, 196.74 ppm. (**2**) ^1^H NMR (CDCl_3_, 400.13 MHz): δ 1.75 (*s*, 6H), 7.81 (*s*, 4H), 10.19 (*s*, 4H), 11.53 (*s*, 2H) ppm. ^13^C NMR (CDCl_3_, 100.62 MHz): δ 30.31, 30.59, 42.08, 123.05, 135.53, 141.31, 162.20, 191.99 ppm; low-resolution mass spectrometry (atmospheric pressure ionization); Thermo Fisher Scientific (ISQ–EC): *m*/*z* [*M*]^+^: calculated = 340.33; measured: 340; and luminescence spectrum (Horiba Jobin Yvon Fluoro­max 3 Spectrofluorimeter): 10^−5^ *M*/aceto­nitrile; λ_exc_ = 356 nm; λ_em_ = 539 nm (full width half maximum = 100 nm).

## Refinement   

Crystal data, data collection and structure refinement details are summarized in Table 3 ▸. The hydrogen atoms for (**1**) and most in (**2**) were refined in a riding-model approximation with C—H = 0.93 or 0.96 Å, *U*_iso_(H) = 1.2*U*_eq_(C) or 1.5*U*_eq_(C_meth­yl_) and O—H = 0.82 Å and *U*_iso_(H) = 1.5*U*_eq_(O). In (**2**), atoms H10, H11, H18, and H5*A* were refined independently with isotropic displacement parameters.

## Supplementary Material

Crystal structure: contains datablock(s) 1, 2. DOI: 10.1107/S2056989018016316/lh5885sup1.cif

Structure factors: contains datablock(s) 1. DOI: 10.1107/S2056989018016316/lh58851sup4.hkl

Structure factors: contains datablock(s) 2. DOI: 10.1107/S2056989018016316/lh58852sup5.hkl

CCDC references: 1879532, 1879531

Additional supporting information:  crystallographic information; 3D view; checkCIF report

## Figures and Tables

**Figure 5:**
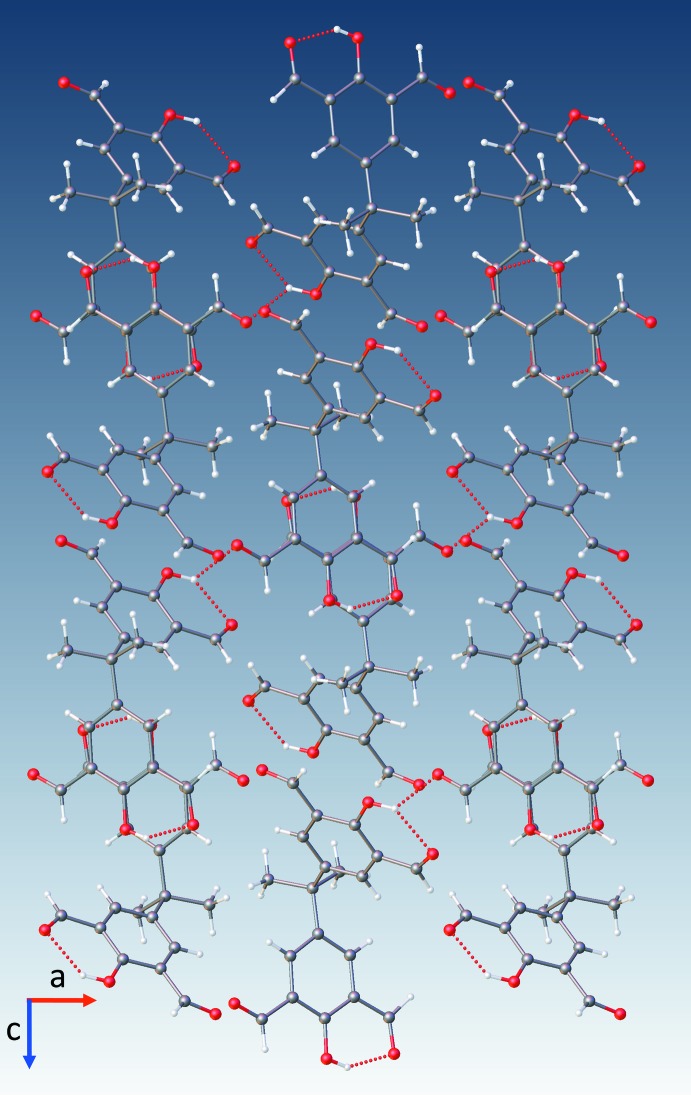
Crystal packing of (**2**) viewed along the *b* axis showing both the intra- and inter­molecular hydrogen bonds (red dashed lines).

**Table 1:** Hydrogen-bond geometry (Å, °) for (**1**)

*D*—H⋯*A*	*D*—H	H⋯*A*	*D*⋯*A*	*D*—H⋯*A*
O2—H2⋯O1	0.82	1.93	2.642 (3)	145
O3—H3⋯O4	0.82	1.90	2.619 (4)	145

**Table 2:** Hydrogen-bond geometry (Å, °) for (**2**)

*D*—H⋯*A*	*D*—H	H⋯*A*	*D*⋯*A*	*D*—H⋯*A*
O2—H2⋯O1	0.82	1.88	2.605 (3)	146
O5—H5*A*⋯O4	0.94 (4)	2.00 (4)	2.745 (3)	135 (3)
O5—H5*A*⋯O3^i^	0.94 (4)	2.14 (4)	2.841 (2)	131 (3)

Symmetry code: (i) 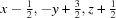.

**Table 3:** Experimental details

	(**1**)	(**2**)
Crystal data		
Chemical formula	C_17_H_16_O_4_	C_19_H_16_O_6_
*M*_r_	284.30	340.32
Crystal system, space group	Monoclinic, *P*2_1_/*c*	Monoclinic, *P*2_1_/*n*
Temperature (K)	298	298
*a*, *b*, *c* (Å)	16.6108 (6), 12.0803 (6), 7.0946 (4)	13.4327 (4), 7.9920 (3), 15.2062 (5)
β (°)	90.396 (4)	90.348 (3)
*V* (Å^3^)	1423.59 (12)	1632.42 (9)
*Z*	4	4
Radiation type	Mo *K*α	Mo *K*α
μ (mm^−1^)	0.09	0.10
Crystal size (mm)	0.35 × 0.3 × 0.28	0.34 × 0.32 × 0.28

Data collection		
Diffractometer	Rigaku Oxford Diffraction SuperNova, Dualflex, EosS2	Rigaku Oxford Diffraction SuperNova, Dualflex, EosS2
Absorption correction	Multi-scan (*CrysAlis PRO*; Bourhis *et al.*, 2015 ▸)	Multi-scan (*CrysAlis PRO*; Bourhis *et al.*, 2015 ▸)
*T*_min_, *T*_max_	0.861, 1.000	0.810, 1.000
No. of measured, independent and observed [*I* > 2σ(*I*)] reflections	6271, 2589, 2036	13629, 3331, 2586
*R*_int_	0.017	0.029
(sin θ/λ)_max_ (Å^−1^)	0.602	0.625

Refinement		
*R*[*F* ^2^ > 2σ(*F* ^2^)], *wR*(*F* ^2^), *S*	0.051, 0.122, 1.10	0.056, 0.167, 1.04
No. of reflections	2589	3331
No. of parameters	195	249
H-atom treatment	H-atom parameters constrained	H atoms treated by a mixture of independent and constrained refinement
Δρ_max_, Δρ_min_ (e Å^−3^)	0.21, −0.20	0.25, −0.27

Computer programs: *CrysAlis PRO* (Rigaku OD, 2015 ▸), *SHELXT* (Sheldrick, 2015*a* ▸), *SHELXL* (Sheldrick, 2015*b* ▸), *OLEX2* (Dolomanov *et al.*, 2009 ▸) and *Mercury* (Macrae *et al.*, 2008 ▸).

## supplementary crystallographic information

### 5,5'-(Propane-2,2-diyl)bis(2-hydroxybenzaldehyde) (1) Crystal data

**Table:**

C_17_H_16_O_4_	*F*(000) = 600
*M**_r_* = 284.30	*D*_x_ = 1.326 Mg m^−^^3^
Monoclinic, *P*2_1_/*c*	Mo *K*α radiation, λ = 0.71073 Å
*a* = 16.6108 (6) Å	Cell parameters from 2453 reflections
*b* = 12.0803 (6) Å	θ = 2.1–25.2°
*c* = 7.0946 (4) Å	µ = 0.09 mm^−^^1^
β = 90.396 (4)°	*T* = 298 K
*V* = 1423.59 (12) Å^3^	Irregular, clear colourless
*Z* = 4	0.35 × 0.3 × 0.28 mm

### 5,5'-(Propane-2,2-diyl)bis(2-hydroxybenzaldehyde) (1) Data collection

**Table:**

Rigaku Oxford Diffraction SuperNova, Dualflex, EosS2 diffractometer	2036 reflections with *I* > 2σ(*I*)
Detector resolution: 8.0945 pixels mm^-1^	*R*_int_ = 0.017
ω scans	θ_max_ = 25.3°, θ_min_ = 2.1°
Absorption correction: multi-scan (CrysAlisPro; Bourhis *et al.*, 2015)	*h* = −20→20
*T*_min_ = 0.861, *T*_max_ = 1.000	*k* = −11→14
6271 measured reflections	*l* = −8→6
2589 independent reflections	

### 5,5'-(Propane-2,2-diyl)bis(2-hydroxybenzaldehyde) (1) Refinement

**Table:**

Refinement on *F*^2^	Hydrogen site location: inferred from neighbouring sites
Least-squares matrix: full	H-atom parameters constrained
*R*[*F*^2^ > 2σ(*F*^2^)] = 0.051	*w* = 1/[σ^2^(*F*_o_^2^) + (0.0347*P*)^2^ + 0.7348*P*] where *P* = (*F*_o_^2^ + 2*F*_c_^2^)/3
*wR*(*F*^2^) = 0.122	(Δ/σ)_max_ < 0.001
*S* = 1.10	Δρ_max_ = 0.21 e Å^−^^3^
2589 reflections	Δρ_min_ = −0.20 e Å^−^^3^
195 parameters	Extinction correction: SHELXL-2016/4 (Sheldrick 2016), Fc^*^=kFc[1+0.001xFc^2^λ^3^/sin(2θ)]^-1/4^
0 restraints	Extinction coefficient: 0.0035 (12)
Primary atom site location: dual	

### 5,5'-(Propane-2,2-diyl)bis(2-hydroxybenzaldehyde) (1) Special details

**Table:**

Geometry. All esds (except the esd in the dihedral angle between two l.s. planes) are estimated using the full covariance matrix. The cell esds are taken into account individually in the estimation of esds in distances, angles and torsion angles; correlations between esds in cell parameters are only used when they are defined by crystal symmetry. An approximate (isotropic) treatment of cell esds is used for estimating esds involving l.s. planes.

### 5,5'-(Propane-2,2-diyl)bis(2-hydroxybenzaldehyde) (1) Fractional atomic coordinates and isotropic or equivalent isotropic displacement parameters (Å^2^)

**Table:**

	*x*	*y*	*z*	*U*_iso_*/*U*_eq_	
O1	0.60832 (9)	0.41639 (19)	0.2405 (3)	0.0801 (6)	
O2	0.52036 (10)	0.26273 (16)	0.0724 (3)	0.0736 (5)	
H2	0.562402	0.293270	0.104187	0.110*	
O3	0.01645 (12)	0.31117 (19)	0.6564 (4)	0.1054 (9)	
H3	−0.028389	0.315525	0.607948	0.158*	
O4	−0.08657 (10)	0.34768 (16)	0.3823 (4)	0.0932 (7)	
C1	0.24872 (11)	0.53761 (17)	0.2163 (3)	0.0461 (5)	
C2	0.26777 (14)	0.6426 (2)	0.3303 (4)	0.0721 (8)	
H2A	0.218770	0.681998	0.354821	0.108*	
H2B	0.303523	0.688954	0.259696	0.108*	
H2C	0.292921	0.622372	0.447559	0.108*	
C3	0.21711 (14)	0.5742 (2)	0.0216 (4)	0.0662 (7)	
H3A	0.199791	0.510478	−0.048481	0.099*	
H3B	0.259284	0.611212	−0.045502	0.099*	
H3C	0.172540	0.623899	0.037436	0.099*	
C4	0.32352 (11)	0.46576 (17)	0.1856 (3)	0.0411 (5)	
C5	0.31596 (13)	0.36340 (18)	0.0949 (3)	0.0482 (5)	
H5	0.264923	0.339291	0.059134	0.058*	
C6	0.38080 (13)	0.29743 (19)	0.0566 (3)	0.0524 (6)	
H6	0.373339	0.230404	−0.005545	0.063*	
C7	0.45731 (12)	0.33011 (19)	0.1102 (3)	0.0486 (5)	
C8	0.46721 (11)	0.43126 (19)	0.2017 (3)	0.0448 (5)	
C9	0.40007 (11)	0.49711 (18)	0.2366 (3)	0.0430 (5)	
H9	0.407386	0.564808	0.296766	0.052*	
C10	0.54638 (13)	0.4685 (2)	0.2607 (3)	0.0600 (7)	
H10	0.550084	0.537495	0.318412	0.072*	
C11	0.18592 (11)	0.47158 (16)	0.3248 (3)	0.0420 (5)	
C12	0.20564 (13)	0.4232 (2)	0.4976 (3)	0.0569 (6)	
H12	0.258393	0.428440	0.541446	0.068*	
C13	0.15019 (15)	0.3682 (2)	0.6053 (4)	0.0699 (8)	
H13	0.165623	0.336218	0.719130	0.084*	
C14	0.07106 (14)	0.36060 (19)	0.5433 (4)	0.0651 (7)	
C15	0.04964 (12)	0.40537 (18)	0.3714 (4)	0.0519 (6)	
C16	0.10733 (12)	0.45953 (18)	0.2658 (3)	0.0469 (5)	
H16	0.092278	0.489091	0.149790	0.056*	
C17	−0.03227 (14)	0.3944 (2)	0.3020 (5)	0.0717 (8)	
H17	−0.044107	0.426019	0.185508	0.086*	

### 5,5'-(Propane-2,2-diyl)bis(2-hydroxybenzaldehyde) (1) Atomic displacement parameters (Å^2^)

**Table:**

	*U*^11^	*U*^22^	*U*^33^	*U*^12^	*U*^13^	*U*^23^
O1	0.0374 (9)	0.1197 (17)	0.0831 (13)	0.0130 (10)	0.0031 (8)	0.0127 (12)
O2	0.0616 (10)	0.0765 (13)	0.0828 (13)	0.0281 (9)	0.0117 (10)	−0.0046 (10)
O3	0.0797 (13)	0.0857 (15)	0.152 (2)	0.0139 (12)	0.0501 (14)	0.0595 (15)
O4	0.0470 (10)	0.0713 (13)	0.162 (2)	−0.0120 (9)	0.0304 (12)	−0.0243 (13)
C1	0.0356 (10)	0.0403 (11)	0.0624 (14)	0.0016 (9)	0.0024 (9)	0.0008 (10)
C2	0.0487 (13)	0.0455 (14)	0.122 (2)	0.0000 (11)	0.0113 (14)	−0.0231 (15)
C3	0.0496 (13)	0.0670 (17)	0.0819 (18)	0.0083 (12)	0.0080 (12)	0.0270 (14)
C4	0.0368 (10)	0.0402 (11)	0.0463 (12)	−0.0006 (9)	0.0021 (8)	0.0001 (9)
C5	0.0441 (11)	0.0461 (13)	0.0543 (13)	−0.0033 (10)	−0.0030 (10)	−0.0045 (10)
C6	0.0619 (14)	0.0427 (12)	0.0527 (13)	0.0055 (11)	0.0038 (11)	−0.0083 (11)
C7	0.0479 (12)	0.0526 (14)	0.0456 (12)	0.0123 (10)	0.0096 (9)	0.0050 (11)
C8	0.0372 (10)	0.0559 (13)	0.0413 (12)	0.0023 (9)	0.0039 (8)	0.0064 (10)
C9	0.0412 (11)	0.0419 (11)	0.0459 (12)	−0.0021 (9)	0.0021 (9)	−0.0013 (9)
C10	0.0406 (12)	0.0845 (18)	0.0551 (14)	−0.0010 (12)	0.0014 (10)	0.0087 (13)
C11	0.0357 (10)	0.0377 (11)	0.0526 (13)	0.0041 (8)	0.0020 (9)	−0.0049 (10)
C12	0.0436 (12)	0.0644 (15)	0.0625 (15)	0.0130 (11)	−0.0022 (11)	0.0059 (12)
C13	0.0669 (16)	0.0731 (18)	0.0698 (17)	0.0226 (13)	0.0113 (13)	0.0274 (14)
C14	0.0550 (14)	0.0443 (14)	0.096 (2)	0.0109 (11)	0.0266 (14)	0.0164 (14)
C15	0.0404 (11)	0.0390 (12)	0.0762 (17)	0.0021 (9)	0.0084 (11)	−0.0042 (12)
C16	0.0408 (11)	0.0457 (12)	0.0543 (13)	0.0015 (9)	0.0011 (9)	−0.0026 (10)
C17	0.0424 (13)	0.0641 (17)	0.109 (2)	−0.0049 (12)	0.0080 (14)	−0.0208 (16)

### 5,5'-(Propane-2,2-diyl)bis(2-hydroxybenzaldehyde) (1) Geometric parameters (Å, º)

**Table:**

O1—C10	1.216 (3)	C5—C6	1.368 (3)
O2—H2	0.8200	C6—H6	0.9300
O2—C7	1.355 (2)	C6—C7	1.381 (3)
O3—H3	0.8200	C7—C8	1.393 (3)
O3—C14	1.354 (3)	C8—C9	1.393 (3)
O4—C17	1.210 (3)	C8—C10	1.449 (3)
C1—C2	1.536 (3)	C9—H9	0.9300
C1—C3	1.539 (3)	C10—H10	0.9300
C1—C4	1.532 (3)	C11—C12	1.395 (3)
C1—C11	1.526 (3)	C11—C16	1.376 (3)
C2—H2A	0.9600	C12—H12	0.9300
C2—H2B	0.9600	C12—C13	1.372 (3)
C2—H2C	0.9600	C13—H13	0.9300
C3—H3A	0.9600	C13—C14	1.386 (4)
C3—H3B	0.9600	C14—C15	1.379 (4)
C3—H3C	0.9600	C15—C16	1.385 (3)
C4—C5	1.399 (3)	C15—C17	1.450 (3)
C4—C9	1.373 (3)	C16—H16	0.9300
C5—H5	0.9300	C17—H17	0.9300
			
C7—O2—H2	109.5	C6—C7—C8	118.96 (19)
C14—O3—H3	109.5	C7—C8—C9	119.45 (19)
C2—C1—C3	107.6 (2)	C7—C8—C10	120.7 (2)
C4—C1—C2	112.22 (17)	C9—C8—C10	119.8 (2)
C4—C1—C3	107.89 (18)	C4—C9—C8	122.5 (2)
C11—C1—C2	107.77 (18)	C4—C9—H9	118.8
C11—C1—C3	111.89 (17)	C8—C9—H9	118.8
C11—C1—C4	109.48 (16)	O1—C10—C8	124.9 (3)
C1—C2—H2A	109.5	O1—C10—H10	117.6
C1—C2—H2B	109.5	C8—C10—H10	117.6
C1—C2—H2C	109.5	C12—C11—C1	120.33 (18)
H2A—C2—H2B	109.5	C16—C11—C1	123.53 (19)
H2A—C2—H2C	109.5	C16—C11—C12	116.1 (2)
H2B—C2—H2C	109.5	C11—C12—H12	118.7
C1—C3—H3A	109.5	C13—C12—C11	122.5 (2)
C1—C3—H3B	109.5	C13—C12—H12	118.7
C1—C3—H3C	109.5	C12—C13—H13	120.2
H3A—C3—H3B	109.5	C12—C13—C14	119.6 (2)
H3A—C3—H3C	109.5	C14—C13—H13	120.2
H3B—C3—H3C	109.5	O3—C14—C13	118.6 (3)
C5—C4—C1	119.71 (17)	O3—C14—C15	121.8 (2)
C9—C4—C1	123.83 (18)	C15—C14—C13	119.5 (2)
C9—C4—C5	116.42 (18)	C14—C15—C16	119.2 (2)
C4—C5—H5	118.7	C14—C15—C17	120.0 (2)
C6—C5—C4	122.5 (2)	C16—C15—C17	120.7 (2)
C6—C5—H5	118.7	C11—C16—C15	123.0 (2)
C5—C6—H6	119.9	C11—C16—H16	118.5
C5—C6—C7	120.2 (2)	C15—C16—H16	118.5
C7—C6—H6	119.9	O4—C17—C15	125.7 (3)
O2—C7—C6	119.0 (2)	O4—C17—H17	117.2
O2—C7—C8	122.0 (2)	C15—C17—H17	117.2
			
O2—C7—C8—C9	180.0 (2)	C5—C6—C7—C8	−0.5 (3)
O2—C7—C8—C10	0.0 (3)	C6—C7—C8—C9	−0.2 (3)
O3—C14—C15—C16	176.9 (2)	C6—C7—C8—C10	179.8 (2)
O3—C14—C15—C17	−3.8 (4)	C7—C8—C9—C4	0.6 (3)
C1—C4—C5—C6	177.4 (2)	C7—C8—C10—O1	−1.6 (4)
C1—C4—C9—C8	−178.02 (19)	C9—C4—C5—C6	−0.5 (3)
C1—C11—C12—C13	176.4 (2)	C9—C8—C10—O1	178.4 (2)
C1—C11—C16—C15	−175.8 (2)	C10—C8—C9—C4	−179.4 (2)
C2—C1—C4—C5	175.9 (2)	C11—C1—C4—C5	56.3 (3)
C2—C1—C4—C9	−6.4 (3)	C11—C1—C4—C9	−126.0 (2)
C2—C1—C11—C12	−67.3 (2)	C11—C12—C13—C14	−0.7 (4)
C2—C1—C11—C16	109.9 (2)	C12—C11—C16—C15	1.6 (3)
C3—C1—C4—C5	−65.7 (2)	C12—C13—C14—O3	−176.5 (2)
C3—C1—C4—C9	112.0 (2)	C12—C13—C14—C15	2.1 (4)
C3—C1—C11—C12	174.6 (2)	C13—C14—C15—C16	−1.6 (4)
C3—C1—C11—C16	−8.2 (3)	C13—C14—C15—C17	177.7 (2)
C4—C1—C11—C12	55.0 (3)	C14—C15—C16—C11	−0.3 (3)
C4—C1—C11—C16	−127.7 (2)	C14—C15—C17—O4	−0.2 (4)
C4—C5—C6—C7	0.9 (3)	C16—C11—C12—C13	−1.1 (3)
C5—C4—C9—C8	−0.3 (3)	C16—C15—C17—O4	179.1 (2)
C5—C6—C7—O2	179.3 (2)	C17—C15—C16—C11	−179.6 (2)

### 5,5'-(Propane-2,2-diyl)bis(2-hydroxybenzaldehyde) (1) Hydrogen-bond geometry (Å, º)

**Table:**

*D*—H···*A*	*D*—H	H···*A*	*D*···*A*	*D*—H···*A*
O2—H2···O1	0.82	1.93	2.642 (3)	145
O3—H3···O4	0.82	1.90	2.619 (4)	145

### 5,5'-(propane-2,2-diyl)bis(2-hydroxyisophthalaldehyde) (2) Crystal data

**Table:**

C_19_H_16_O_6_	*F*(000) = 712
*M**_r_* = 340.32	*D*_x_ = 1.385 Mg m^−^^3^
Monoclinic, *P*2_1_/*n*	Mo *K*α radiation, λ = 0.71073 Å
*a* = 13.4327 (4) Å	Cell parameters from 4266 reflections
*b* = 7.9920 (3) Å	θ = 2.0–26.0°
*c* = 15.2062 (5) Å	µ = 0.10 mm^−^^1^
β = 90.348 (3)°	*T* = 298 K
*V* = 1632.42 (9) Å^3^	Irregular, yellow
*Z* = 4	0.34 × 0.32 × 0.28 mm

### 5,5'-(propane-2,2-diyl)bis(2-hydroxyisophthalaldehyde) (2) Data collection

**Table:**

Rigaku Oxford Diffraction SuperNova, Dualflex, EosS2 diffractometer	3331 independent reflections
Radiation source: fine-focus sealed X-ray tube, Enhance (Mo) X-ray Source	2586 reflections with *I* > 2σ(*I*)
Graphite monochromator	*R*_int_ = 0.029
Detector resolution: 8.0945 pixels mm^-1^	θ_max_ = 26.4°, θ_min_ = 2.0°
ω scans	*h* = −16→16
Absorption correction: multi-scan (CrysAlisPro; Bourhis *et al.*, 2015)	*k* = −8→9
*T*_min_ = 0.810, *T*_max_ = 1.000	*l* = −19→19
13629 measured reflections	

### 5,5'-(propane-2,2-diyl)bis(2-hydroxyisophthalaldehyde) (2) Refinement

**Table:**

Refinement on *F*^2^	Primary atom site location: dual
Least-squares matrix: full	Hydrogen site location: mixed
*R*[*F*^2^ > 2σ(*F*^2^)] = 0.056	H atoms treated by a mixture of independent and constrained refinement
*wR*(*F*^2^) = 0.167	*w* = 1/[σ^2^(*F*_o_^2^) + (0.085*P*)^2^ + 0.540*P*] where *P* = (*F*_o_^2^ + 2*F*_c_^2^)/3
*S* = 1.04	(Δ/σ)_max_ < 0.001
3331 reflections	Δρ_max_ = 0.25 e Å^−^^3^
249 parameters	Δρ_min_ = −0.27 e Å^−^^3^
0 restraints	

### 5,5'-(propane-2,2-diyl)bis(2-hydroxyisophthalaldehyde) (2) Special details

**Table:**

Geometry. All esds (except the esd in the dihedral angle between two l.s. planes) are estimated using the full covariance matrix. The cell esds are taken into account individually in the estimation of esds in distances, angles and torsion angles; correlations between esds in cell parameters are only used when they are defined by crystal symmetry. An approximate (isotropic) treatment of cell esds is used for estimating esds involving l.s. planes.

### 5,5'-(propane-2,2-diyl)bis(2-hydroxyisophthalaldehyde) (2) Fractional atomic coordinates and isotropic or equivalent isotropic displacement parameters (Å^2^)

**Table:**

	*x*	*y*	*z*	*U*_iso_*/*U*_eq_	
C1	0.56797 (16)	0.2277 (3)	0.75859 (13)	0.0451 (5)	
C2	0.5020 (3)	0.0866 (3)	0.79457 (17)	0.0744 (8)	
H2A	0.433239	0.115432	0.786234	0.112*	
H2B	0.515438	0.071710	0.856159	0.112*	
H2C	0.516230	−0.015436	0.763805	0.112*	
C3	0.67756 (19)	0.1817 (3)	0.77738 (16)	0.0650 (7)	
H3A	0.719548	0.274519	0.762469	0.098*	
H3B	0.695707	0.086163	0.742724	0.098*	
H3C	0.685618	0.155720	0.838652	0.098*	
C4	0.55349 (14)	0.2495 (2)	0.65879 (12)	0.0398 (4)	
C5	0.62192 (14)	0.3416 (3)	0.61068 (13)	0.0425 (5)	
H5	0.674345	0.392670	0.640554	0.051*	
C6	0.61570 (15)	0.3610 (3)	0.51995 (13)	0.0442 (5)	
C7	0.53582 (16)	0.2880 (3)	0.47460 (13)	0.0477 (5)	
C8	0.46395 (15)	0.2017 (3)	0.52114 (14)	0.0470 (5)	
C9	0.47444 (15)	0.1818 (3)	0.61219 (14)	0.0458 (5)	
H9	0.426579	0.120883	0.642407	0.055*	
C10	0.69131 (19)	0.4569 (3)	0.47277 (17)	0.0598 (6)	
H10	0.683 (2)	0.462 (3)	0.4070 (19)	0.073 (8)*	
C11	0.37722 (19)	0.1293 (4)	0.4765 (2)	0.0669 (7)	
H11	0.324 (2)	0.068 (3)	0.5214 (17)	0.065 (7)*	
C12	0.53648 (14)	0.3898 (3)	0.80494 (12)	0.0400 (5)	
C13	0.44519 (15)	0.4626 (3)	0.78465 (13)	0.0449 (5)	
H13	0.407023	0.415913	0.739712	0.054*	
C14	0.40839 (15)	0.6016 (3)	0.82828 (13)	0.0460 (5)	
C15	0.46436 (15)	0.6737 (3)	0.89671 (13)	0.0421 (5)	
C16	0.55783 (14)	0.6066 (3)	0.91700 (13)	0.0426 (5)	
C17	0.59223 (14)	0.4663 (3)	0.87119 (12)	0.0422 (5)	
H17	0.654434	0.422634	0.885479	0.051*	
C18	0.31154 (19)	0.6673 (4)	0.80118 (17)	0.0662 (7)	
H18	0.277 (2)	0.595 (4)	0.755 (2)	0.087 (9)*	
C19	0.61762 (17)	0.6814 (3)	0.98792 (16)	0.0571 (6)	
H19	0.5921 (19)	0.790 (3)	1.0142 (17)	0.067 (7)*	
O1	0.36389 (14)	0.1404 (3)	0.39694 (14)	0.0851 (7)	
O2	0.53075 (14)	0.3050 (3)	0.38619 (10)	0.0706 (5)	
H2	0.479652	0.260776	0.367754	0.106*	
O3	0.75935 (14)	0.5285 (3)	0.50684 (12)	0.0779 (6)	
O4	0.27133 (14)	0.7886 (3)	0.83289 (13)	0.0869 (7)	
O5	0.43151 (13)	0.8023 (2)	0.94557 (11)	0.0565 (4)	
H5A	0.366 (3)	0.834 (4)	0.932 (2)	0.096 (10)*	
O6	0.69410 (13)	0.6221 (3)	1.01611 (13)	0.0749 (6)	

### 5,5'-(propane-2,2-diyl)bis(2-hydroxyisophthalaldehyde) (2) Atomic displacement parameters (Å^2^)

**Table:**

	*U*^11^	*U*^22^	*U*^33^	*U*^12^	*U*^13^	*U*^23^
C1	0.0571 (12)	0.0433 (12)	0.0349 (10)	0.0031 (9)	−0.0028 (8)	0.0021 (9)
C2	0.119 (2)	0.0514 (15)	0.0532 (14)	−0.0140 (15)	0.0114 (14)	0.0062 (12)
C3	0.0797 (16)	0.0667 (16)	0.0484 (13)	0.0329 (13)	−0.0166 (12)	−0.0045 (11)
C4	0.0436 (10)	0.0397 (11)	0.0362 (10)	0.0046 (8)	−0.0029 (8)	−0.0034 (8)
C5	0.0428 (10)	0.0440 (11)	0.0405 (11)	−0.0006 (9)	−0.0066 (8)	−0.0020 (9)
C6	0.0460 (10)	0.0449 (12)	0.0417 (11)	0.0012 (9)	−0.0013 (8)	0.0007 (9)
C7	0.0560 (12)	0.0477 (12)	0.0392 (11)	0.0097 (10)	−0.0085 (9)	−0.0048 (9)
C8	0.0427 (10)	0.0479 (12)	0.0503 (12)	0.0050 (9)	−0.0095 (9)	−0.0094 (10)
C9	0.0422 (10)	0.0467 (12)	0.0486 (12)	−0.0006 (9)	0.0004 (9)	−0.0036 (9)
C10	0.0656 (14)	0.0639 (16)	0.0498 (13)	−0.0071 (12)	−0.0004 (11)	0.0063 (12)
C11	0.0536 (13)	0.0721 (17)	0.0746 (18)	0.0058 (12)	−0.0217 (13)	−0.0220 (14)
C12	0.0458 (10)	0.0433 (11)	0.0309 (9)	0.0013 (8)	0.0006 (8)	0.0046 (8)
C13	0.0458 (10)	0.0547 (13)	0.0343 (10)	0.0009 (9)	−0.0045 (8)	−0.0014 (9)
C14	0.0433 (10)	0.0585 (13)	0.0362 (10)	0.0051 (9)	−0.0001 (8)	0.0039 (9)
C15	0.0458 (10)	0.0432 (11)	0.0374 (10)	−0.0001 (9)	0.0060 (8)	0.0037 (8)
C16	0.0427 (10)	0.0480 (12)	0.0373 (10)	−0.0043 (9)	0.0007 (8)	0.0004 (9)
C17	0.0395 (9)	0.0497 (12)	0.0373 (10)	0.0015 (9)	−0.0003 (8)	0.0034 (9)
C18	0.0561 (13)	0.0870 (19)	0.0555 (14)	0.0234 (13)	−0.0084 (11)	−0.0153 (14)
C19	0.0472 (12)	0.0662 (16)	0.0578 (14)	−0.0025 (11)	−0.0040 (10)	−0.0106 (12)
O1	0.0752 (12)	0.1010 (15)	0.0786 (13)	0.0110 (11)	−0.0387 (10)	−0.0277 (11)
O2	0.0875 (13)	0.0870 (14)	0.0371 (8)	0.0030 (10)	−0.0139 (8)	−0.0001 (8)
O3	0.0766 (12)	0.0875 (14)	0.0697 (12)	−0.0279 (11)	−0.0005 (9)	0.0071 (10)
O4	0.0695 (11)	0.1126 (16)	0.0784 (13)	0.0418 (11)	−0.0131 (10)	−0.0226 (12)
O5	0.0581 (9)	0.0565 (10)	0.0550 (9)	0.0090 (8)	0.0009 (7)	−0.0105 (8)
O6	0.0604 (10)	0.0890 (14)	0.0753 (12)	0.0004 (9)	−0.0174 (9)	−0.0135 (10)

### 5,5'-(propane-2,2-diyl)bis(2-hydroxyisophthalaldehyde) (2) Geometric parameters (Å, º)

**Table:**

C1—C2	1.537 (3)	C12—C1	1.536 (3)
C1—C3	1.542 (3)	C12—C17	1.393 (3)
C2—H2A	0.9600	C13—C12	1.390 (3)
C2—H2B	0.9600	C13—H13	0.9300
C2—H2C	0.9600	C13—C14	1.386 (3)
C3—H3A	0.9600	C14—C18	1.460 (3)
C3—H3B	0.9600	C15—C14	1.404 (3)
C3—H3C	0.9600	C16—C15	1.398 (3)
C4—C1	1.539 (3)	C16—C19	1.468 (3)
C4—C5	1.389 (3)	C17—C16	1.400 (3)
C4—C9	1.383 (3)	C17—H17	0.9300
C5—H5	0.9300	C18—H18	1.02 (3)
C5—C6	1.390 (3)	C19—H19	1.02 (3)
C6—C7	1.399 (3)	O1—C11	1.225 (3)
C6—C10	1.464 (3)	O2—C7	1.353 (3)
C7—C8	1.384 (3)	O2—H2	0.8200
C8—C9	1.400 (3)	O3—C10	1.194 (3)
C8—C11	1.464 (3)	O4—C18	1.211 (3)
C9—H9	0.9300	O5—C15	1.344 (3)
C10—H10	1.01 (3)	O5—H5A	0.94 (3)
C11—H11	1.10 (3)	O6—C19	1.208 (3)
			
C2—C1—C3	108.1 (2)	C4—C9—H9	118.8
C2—C1—C4	111.37 (18)	C8—C9—H9	118.8
C4—C1—C3	108.94 (17)	C6—C10—H10	115.5 (16)
C12—C1—C2	107.15 (18)	O3—C10—C6	124.8 (2)
C12—C1—C3	112.43 (17)	O3—C10—H10	119.8 (16)
C12—C1—C4	108.90 (16)	C8—C11—H11	113.7 (13)
C1—C2—H2A	109.5	O1—C11—C8	122.7 (3)
C1—C2—H2B	109.5	O1—C11—H11	123.6 (13)
C1—C2—H2C	109.5	C13—C12—C1	119.75 (17)
H2A—C2—H2B	109.5	C13—C12—C17	116.53 (19)
H2A—C2—H2C	109.5	C17—C12—C1	123.62 (17)
H2B—C2—H2C	109.5	C12—C13—H13	118.5
C1—C3—H3A	109.5	C14—C13—C12	123.06 (18)
C1—C3—H3B	109.5	C14—C13—H13	118.5
C1—C3—H3C	109.5	C13—C14—C15	119.53 (18)
H3B—C3—H3A	109.5	C13—C14—C18	118.2 (2)
H3B—C3—H3C	109.5	C15—C14—C18	122.3 (2)
H3C—C3—H3A	109.5	C16—C15—C14	118.81 (19)
C5—C4—C1	119.94 (17)	O5—C15—C14	123.20 (19)
C9—C4—C1	123.58 (18)	O5—C15—C16	117.97 (19)
C9—C4—C5	116.48 (18)	C15—C16—C17	119.76 (18)
C4—C5—H5	118.5	C15—C16—C19	119.5 (2)
C4—C5—C6	123.06 (18)	C17—C16—C19	120.73 (19)
C6—C5—H5	118.5	C12—C17—C16	122.25 (18)
C5—C6—C7	118.93 (19)	C12—C17—H17	118.9
C5—C6—C10	120.46 (19)	C16—C17—H17	118.9
C7—C6—C10	120.6 (2)	C14—C18—H18	113.3 (17)
C8—C7—C6	119.40 (18)	O4—C18—C14	125.1 (2)
O2—C7—C6	118.8 (2)	O4—C18—H18	121.4 (17)
O2—C7—C8	121.82 (19)	C16—C19—H19	116.9 (14)
C7—C8—C9	119.73 (18)	O6—C19—C16	124.2 (2)
C7—C8—C11	121.0 (2)	O6—C19—H19	119.0 (15)
C9—C8—C11	119.3 (2)	C7—O2—H2	109.5
C4—C9—C8	122.31 (19)	C15—O5—H5A	113 (2)
			
C1—C4—C5—C6	177.43 (18)	C12—C13—C14—C18	179.9 (2)
C1—C4—C9—C8	−179.08 (19)	C12—C17—C16—C15	0.2 (3)
C1—C12—C17—C16	−174.81 (18)	C12—C17—C16—C19	178.5 (2)
C4—C5—C6—C7	1.5 (3)	C13—C12—C1—C2	−71.0 (2)
C4—C5—C6—C10	−178.8 (2)	C13—C12—C1—C3	170.37 (19)
C5—C4—C1—C2	−164.9 (2)	C13—C12—C1—C4	49.6 (2)
C5—C4—C1—C3	−45.8 (3)	C13—C12—C17—C16	1.6 (3)
C5—C4—C1—C12	77.2 (2)	C13—C14—C18—O4	−179.1 (3)
C5—C4—C9—C8	1.0 (3)	C14—C13—C12—C1	175.04 (19)
C5—C6—C7—C8	1.5 (3)	C14—C13—C12—C17	−1.5 (3)
C5—C6—C7—O2	−179.06 (19)	C15—C14—C18—O4	1.2 (4)
C5—C6—C10—O3	−2.9 (4)	C15—C16—C19—O6	172.1 (2)
C6—C7—C8—C9	−3.0 (3)	C16—C15—C14—C13	2.2 (3)
C6—C7—C8—C11	177.5 (2)	C16—C15—C14—C18	−178.1 (2)
C7—C6—C10—O3	176.9 (2)	C17—C12—C1—C2	105.3 (2)
C7—C8—C9—C4	1.8 (3)	C17—C12—C1—C3	−13.3 (3)
C7—C8—C11—O1	0.3 (4)	C17—C12—C1—C4	−134.15 (19)
C9—C4—C1—C2	15.2 (3)	C17—C16—C15—C14	−2.2 (3)
C9—C4—C1—C3	134.4 (2)	C17—C16—C15—O5	176.20 (18)
C9—C4—C1—C12	−102.7 (2)	C17—C16—C19—O6	−6.2 (4)
C9—C4—C5—C6	−2.7 (3)	C19—C16—C15—C14	179.5 (2)
C9—C8—C11—O1	−179.2 (2)	C19—C16—C15—O5	−2.1 (3)
C10—C6—C7—C8	−178.3 (2)	O2—C7—C8—C9	177.5 (2)
C10—C6—C7—O2	1.2 (3)	O2—C7—C8—C11	−1.9 (3)
C11—C8—C9—C4	−178.8 (2)	O5—C15—C14—C13	−176.02 (19)
C12—C13—C14—C15	−0.4 (3)	O5—C15—C14—C18	3.6 (3)

### 5,5'-(propane-2,2-diyl)bis(2-hydroxyisophthalaldehyde) (2) Hydrogen-bond geometry (Å, º)

**Table:**

*D*—H···*A*	*D*—H	H···*A*	*D*···*A*	*D*—H···*A*
O2—H2···O1	0.82	1.88	2.605 (3)	146
O5—H5*A*···O4	0.94 (4)	2.00 (4)	2.745 (3)	135 (3)
O5—H5*A*···O3^i^	0.94 (4)	2.14 (4)	2.841 (2)	131 (3)

Symmetry code: (i) *x*−1/2, −*y*+3/2, *z*+1/2.
